# Why Do Patients Bleed?

**DOI:** 10.1055/s-0036-1579657

**Published:** 2016-02-24

**Authors:** Jennifer Curnow, Leonardo Pasalic, Emmanuel J. Favaloro

**Affiliations:** 1 Department of Clinical and Laboratory Hematology, Institute of Clinical Pathology and Medical Research and Westmead Hospital, Sydney Centres for Thrombosis and Hemostasis, Westmead, Australia; 2 Pathology West, NSW Health Pathology, Westmead, Australia

**Keywords:** surgery, bleeding, hemostasis, platelet function, coagulation

## Abstract

Patients undergoing surgical procedures can bleed for a variety of reasons. Assuming that the surgical procedure has progressed well and that the surgeon can exclude surgical reasons for the unexpected bleeding, then the bleeding may be due to structural (anatomical) anomalies or disorders, recent drug intake, or disorders of hemostasis, which may be acquired or congenital. The current review aims to provide an overview of reasons that patients bleed in the perioperative setting, and it also provides guidance on how to screen for these conditions, through consideration of appropriate patient history and examination prior to surgical intervention, as well as guidance on investigating and managing the cause of unexpected bleeding.

Patients undergoing surgical procedures can bleed for one or more reasons (summarized in Table 1 ). Assuming that the surgical procedure has progressed well and that the surgeon can exclude surgical reasons for the unexpected bleeding, then the bleeding may be due to structural (anatomical) anomalies or disorders, recent drug intake, or disorders of hemostasis, which in turn can be acquired or congenital. This article primarily aims to overview the process of hemostasis and show how deranged hemostasis, whether due to acquired events (including drugs, supplements, among others) or congenital disorders/deficiencies, can give rise to unexpected bleeding. This narrative review also provides guidance on how to screen for these conditions, through consideration of appropriate patient history and examination prior to surgical intervention, as well as providing guidance on investigating and managing the cause of bleeding.

**Table 1:** A summary of why patients may bleed as a result of surgical procedures

Why a patient may bleed	Comments
The surgical intervention itself, including anomalous vasculature and anatomical anomalies	Surgeons may be reticent to consider this factor, but it is the known cause of most unexpected bleeds and should be evaluated before/concurrent to investigation of other possible causes of bleeding; importantly, subtle differences may exist in the anatomy of individual patients
Tissue/collagen disorders	Rare; see Table 2 for a list
Drug-related causes	Antithrombotic/anticoagulant/antiplatelet agents (refer to Table 3 ) Unintended consequences of many supplements Other drugs
Other acquired disorders	Liver disease, vitamin K deficiency, renal failure with uremia, bone marrow failure due to hematological disorders or chemotherapy treatment Immune thrombocytopenic purpura Disseminated intravascular coagulation Factor inhibitors
Congenital disorders	Platelet disorders (primary hemostasis) von Willebrand disease (primary and secondary hemostasis) Hemophilias (deficiencies in factors VIII or IX or XI; secondary hemostasis) Rare bleeding disorders (e.g., defects/deficiencies in fibrinogen, and factors II, V, VII, X, XIII; usually secondary hemostasis)

## What Is Hemostasis?

**Fig. 1:**
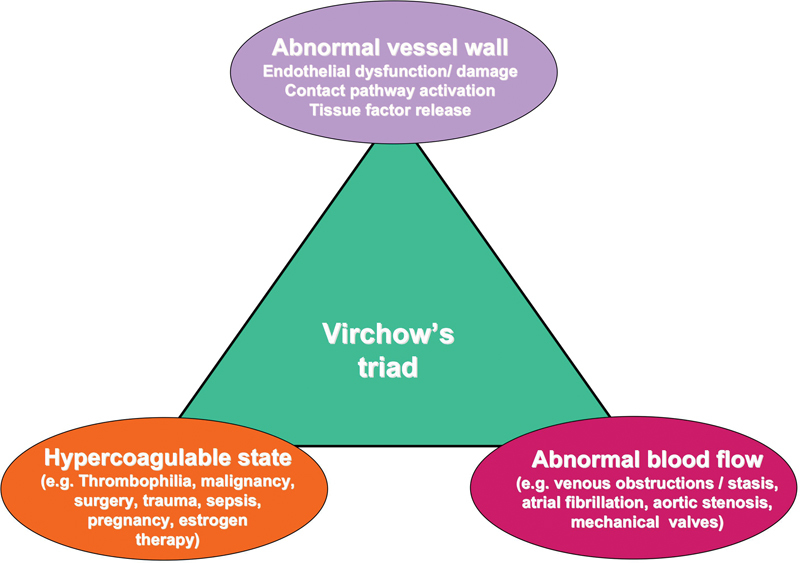
Virchow's triad. Although this visual describes the basic components contributing to thrombosis, analogous considerations around these concepts can also be applied to bleeding, albeit in the “opposite direction” to thrombosis.

The function of normal hemostasis is to maintain intravascular blood in a fluid state while responding to injury by forming a localized clot to prevent further bleeding and subsequently to remove the thrombus to permit wound healing. 1 Optimal hemostasis requires a balance between the processes involved in activation (prohemostatic/procoagulant) and inhibition (antihemostatic/anticoagulant). Components that lead to deranged thrombosis essentially comprise those identified by Virchow's triad ( Fig. 1 ). A similar conceptual approach, albeit in the other direction, can be used to describe deranged hemostasis leading to bleeding. The basic components of hemostasis comprise the blood vessel wall (which in turn includes the endothelial cell lining and the subendothelial matrix components such as collagen), the blood components (especially platelets, coagulation factors, and other adhesive proteins), plus various inhibitors and the fibrinolytic system. Intact endothelium has several properties that essentially inhibit thrombus formation in normally flowing blood. 2 Endothelium is covered by glycocalyx, which contains heparan sulfate, an activator of antithrombin (in turn an inhibitor of hemostasis). Endothelium also expresses thrombomodulin, which binds free thrombin (a potent procoagulant protein) and ADPase. ADPase degrades the platelet agonist adenosine diphosphate (which might otherwise activate platelets). Endothelium also produces tissue plasminogen activator (a protein involved in the breakdown of clots) as well as the vasodilators nitric oxide and prostacyclin. 1 3 4 Blood vessels constrict in response to injury by smooth muscle contraction, and the microvasculature vasoconstricts in response to endothelin released from endothelial cells and thromboxane released from activated platelets. 1 5 6

When injury occurs, platelets adhere to von Willebrand factor (VWF) in the exposed subendothelial matrix, via the platelet glycoprotein (Gp) Ib-V-IX receptor. This initial adhesion occurs under high shear flow conditions and is reversible. However, adhesion also triggers platelet activation via GPIIb/IIIa receptor activation, platelet granule release, phospholipid exposure, shedding of microparticles, shape change with formation of pseudopodia, and irreversible binding to matrix ligands, under low shear. 7 Activated platelet GPIIb/IIIa then mediates platelet aggregation via fibrinogen and VWF. Release of the platelet granule contents and microparticles recruits additional platelets. 1 8 9 This process is often termed *primary hemostasis* .

**Fig. 2:**
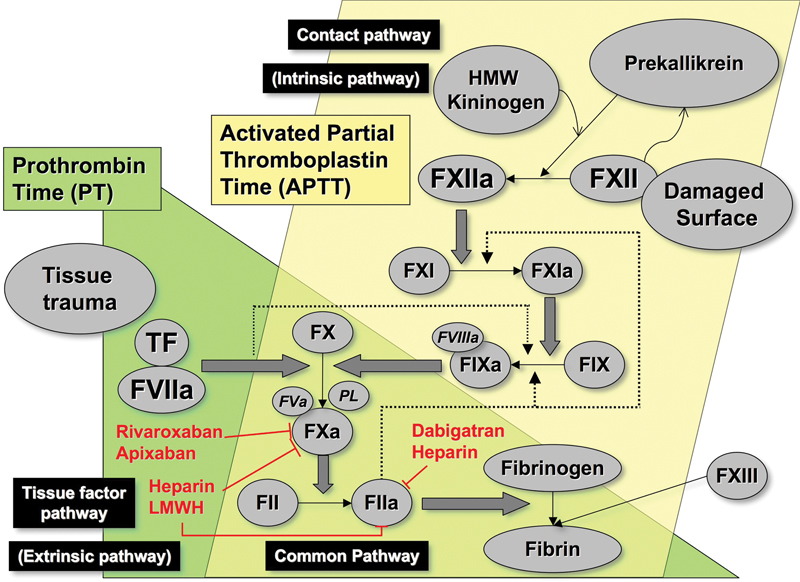
The concept of intrinsic, extrinsic, and common pathways of coagulation, as representative of the waterfall or cascade models of coagulation, and their relationship to the common coagulation assays. In these assays, reagents contain tissue extracts or recombinant TF (for PT) to mimic the extrinsic pathway and various activators (e.g., ellagic acid, micronized silica) to mimic a damaged surface (for APTT) to imitate the contact or intrinsic pathway. Also shown in this figure are some common anticoagulant control points. The vitamin K antagonists (including warfarin) affect factors II, VII, IX and X. Abbreviations: APTT, activated partial thromboplastin time; F, factor; HMW, high molecular weight; LMWH, low molecular weight heparin; PL, phospholipid; PT, prothrombin time; TF, tissue factor.

*Secondary hemostasis* comprises additional processes of coagulation (clotting) and fibrinolysis and can be initiated either directly or by primary hemostasis. 1 Historically, secondary hemostasis was envisaged as a waterfall or cascade model, as initially proposed in 1964 by Davie and Ratnoff and by Macfarlane. 10 11 This model described the circulation of coagulation proteins as inactive zymogens, which in the presence of phospholipid and calcium underwent sequential activation to serine proteases. The theory explained the roles of all the known coagulation factors and correlated well with the observations from laboratory testing, especially the plasma-based activated partial thromboplastin time (APTT) and prothrombin time (PT) assays ( Fig. 2 ). 12

**Fig. 3:**
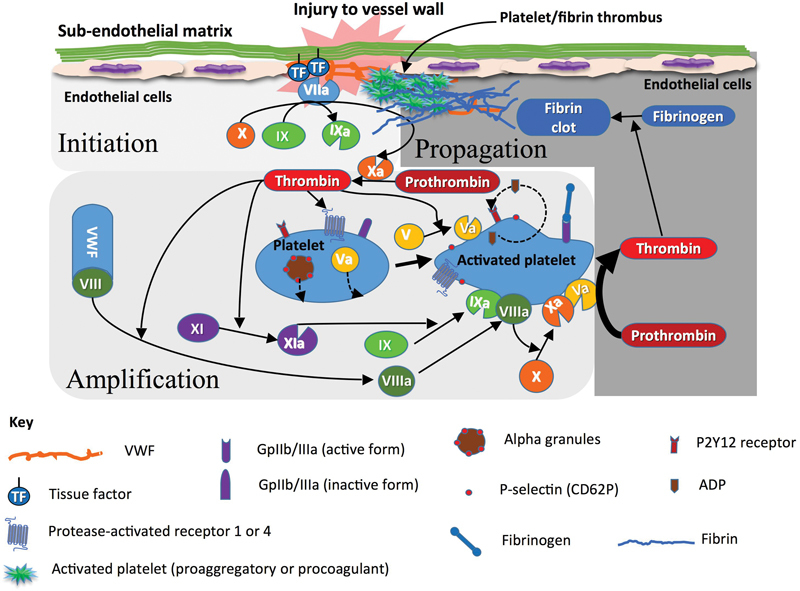
The cell-based model of coagulation and the concepts of initiation, priming/amplification, and propagation of thrombin generation. Potential defects in primary or secondary hemostasis, as depicted in this figure, can be associated with bleeding.

Our current understanding of this process revolves around a cell-based model in which the process of coagulation is initiated by tissue factor (TF). 8 12 13 In this model, the coagulation process is regulated by cell surface properties, and there are specific cellular receptors for the coagulation proteins, as well as the exposure of negatively charged phospholipids. Three overlapping, rather than sequential, phases of coagulation have now been described: initiation, priming (or amplification), and propagation ( Fig. 3 ). TF, the primary coagulation trigger in this model, may be derived from the surface of extravascular cells, such as fibroblasts within the vessel wall or from blood-borne microparticles, and can also be expressed by stimulated endothelial cells and monocytes, as well as variety of other sources. 14 15 16

During *initiation* , TF binds to factor VIIa (FVIIa) and activates a small amount of factor IX (FIX) and factor X (FX) directly. At its site of production on the TF-bearing cell, factor Xa (FXa) activates factor V and then complexes with factor Va (FVa), forming the prothrombinase complex, which converts a small amount of prothrombin to thrombin locally. 8 12

In the *priming/amplification* phase, the small amount of thrombin produced acts on protease-activated receptors to further activate platelets, which release the contents of α granules, including factor FV (FV). 17 Thrombin cleaves factor VIII (FVIII), leading to its dissociation from VWF, and activates it along with FV and factor XI (FXI), and tissue factor pathway inhibitor inactivates the FVIIa/TF/FXa complex. 8 12

During *propagation* , the FIXa produced by TF/FVIIa binds to activated platelets and further FIXa is generated on the platelet surface by FXIa. The tenase complex of FVIIIa/FIXa activates FX, which then complexes with FVa, generating a significant thrombin burst. 12 17 The main aspects of this process are summarized in Fig. 3 . According to this model, in the absence of FVIII, FIX, or FXI, this amplification and clot propagation are impaired, even though TF still initiates the process. However, the absence of contact activating factors (including factor XII [FXII]) does not impair the coagulation process, which helps explain why deficiencies of FVIII, FIX, and factor XI (FXI) can lead to bleeding but why deficiencies of the contact factors such as FXII do not.

The generated thrombin binds to fibrinogen and cleaves it, releasing fibrinopeptides A and B, which then polymerize by forming protofibrils with adjacent fibrin molecules. Thrombin also activates factor XIII (FXIII), which stabilizes the fibrin clot by forming cross-links ( Fig. 2 ). Fibrin networks with tightly cross-linked fibers are less permeable and more resistant to fibrinolysis, 18 19 which helps to explain why FXIII deficiency is also associated with bleeding.

Fibrinolytic components are localized on the surface of a fibrin clot to mediate fibrin degradation. To facilitate this action, the efficiency of tissue-type plasminogen activator is enhanced in the presence of fibrin. 20 Tissue-type plasminogen activator cleaves plasminogen to the active enzyme plasmin, which produces fibrin degradation products. The fibrinolytic system is tightly controlled by inhibitors. Alpha 2-antiplasmin binds to plasmin to inactivate it, and plasminogen activator inhibitor (PAI-1) complexes with tissue-type plasminogen activator to prevent further plasmin production. In addition, in the presence of thrombomodulin, thrombin activates a further inhibitor of fibrinolysis (thrombin activatable fibrinolysis inhibitor [TAFI]), thus linking the coagulation system with fibrinolysis. TAFI reduces plasminogen binding to the fibrin clot by cleaving the C-terminal lysine and arginine residues from fibrin. 21

The coagulation pathway is also regulated by inhibitors. In addition to tissue factor pathway inhibitor, which binds the TF/VIIa/Xa complex generated during the initiation phase of coagulation, there are two other major inhibitory mechanisms. Antithrombin inhibits the serine proteases thrombin FXa and to a lesser extent factor XIa (FXIa) and factor IXa (FIXa). The activity of antithrombin is greatly enhanced by heparan sulfate, which also localizes antithrombin action to the endothelial cell surface. The final inhibitory mechanism involves activated protein C, which is aided by its cofactor protein S and cleaves FVIIIa and FVa to inactivate them. Circulating protein C is bound to the endothelial cell by a specific receptor, bringing it into close proximity with the thrombin/thrombomodulin complex, which activates protein C. 22 Consequently, the inhibitors are also localized at the site of injury and clot formation.

The current model of coagulation emphasizes the central role of thrombin. Thrombin is not merely the enzyme converting fibrinogen to fibrin. It also activates platelet aggregation, endothelium, leukocytes, and factors V, VIII, IX, XI, and XIII, and it triggers activation of the antithrombotic protein C pathway and inhibition of fibrinolysis via TAFI. 23 24

Thrombin is also involved in the innate immune system and amplifies and modifies inflammatory responses. 25 The complement system has direct effects on coagulation and interacts with inflammatory mediators. 26 These effects of thrombin may also be relevant in hypercoagulable states.

Although it is convenient to separate primary and secondary hemostasis in terms of models, the two systems are intricately linked. For example, VWF is a large and complex protein synthesized in endothelial cells and megakaryocytes, 27 28 and it contributes to both primary and secondary hemostasis. VWF binds to platelets, FVIII, and subendothelial matrix components such as collagen. VWF is thus able to deliver both FVIII and platelets, then localize these to sites of tissue injury, and also further facilitate the adhesion of platelets to each other and to collagen in the subendothelial. 29 30 31 Platelets also contain an armamentarium of procoagulant material, including FV, that are delivered and released to the area of tissue injury, as well as expressing many cell surface adhesion molecules that also bind to collagen and fibrinogen in addition to VWF.

As already noted, the entire hemostasis system is tightly controlled by various checks and balances. An inherited or acquired defect in the system may consequently overwhelm the inbuilt compensatory processes and lead to uncontrolled bleeding or thrombosis. There are many potential deficiencies or defects now recognized within hemostasis ( Table 2 ), including defects of platelets and clotting factors and VWF. These may lead to platelet disorders, hemophilia, rare bleeding disorders, and von Willebrand disease (VWD), just to name a few such disorders.

**Table 2:** Classification of hereditary and acquired disorders of hemostasis and mechanisms

	Mechanism
Primary hemostasis	
Hereditary diseases	
von Willebrand disease	Impaired platelet adhesion/aggregation
Bernard-Soulier syndrome	Impaired platelet adhesion/aggregation
Glanzmann thrombasthenia	Impaired platelet adhesion/aggregation and Thrombocytopenia
Platelet storage pool disease	Impaired platelet adhesion/aggregation
Gray platelet syndrome	Impaired platelet adhesion/aggregation
May-Hegglin anomaly	Macrothrombocytopenia
Wiskott-Aldrich syndrome	Microthrombocytopenia
Acquired disorders	
Immune thrombocytopenic purpura	Thrombocytopenia
Drug-induced immune thrombocytopenia (e.g., quinine, sulfonamides)	Thrombocytopenia
Drug-induced platelet dysfunction (e.g., antiplatelet therapies, NSAIDs)	Impaired adhesion and/or aggregation
Mechanical platelet destruction (e.g., cardiac bypass)	Thrombocytopenia
Disseminated intravascular coagulation	Thrombocytopenia and secondary hemostatic defects, including hyperfibrinolysis
Renal failure with uremia	Impaired platelet aggregation and secretion
Liver disease	Rebalanced hemostasis with multiple abnormalities
Secondary hemostasis	
Hereditary diseases	
Hemophilia A (factor VIII deficiency)	Impaired coagulation
Hemophilia B (factor IX deficiency)	Impaired coagulation
Rare bleeding disorders: deficiencies in factors II, V, VII, X, XI	Impaired coagulation
Factor XIII deficiency	Impaired coagulation
Afibrinogenemia/hypofibrinogenemia	Impaired coagulation
nherited dysfibrinogenemias	Impaired coagulation
Ehlers-Danlos syndrome	Connective tissue disorder
Hereditary hemorrhagic telangiectasia	Connective tissue disorder
Acquired disorders	
Acquired inhibitors of specific coagulation factors	Impaired coagulation
Vitamin K deficiency	Impaired coagulation
Afibrinogenemia/hypofibrinogenemia (DIC)	Impaired coagulation
Hyperfibrinolysis	Increased fibrinolysis
Vitamin C deficiency (scurvy)	Connective tissue disorder
Anticoagulant ingestion	Impaired coagulation
Liver disease	Rebalanced hemostasis with multiple abnormalities

Abbreviations: DIC, disseminated intravascular coagulation; NSAIDs, nonsteroidal anti-inflammatory drugs.

## How Does Surgery Affect the Process of Hemostasis?

Surgery and the associated tissue trauma result in various acute changes in the hemostatic system aimed at increasing clot formation and thus minimizing blood loss. Surgical incisions release TF from endothelial and other tissue sources, initiating the process of coagulation. If inflammation or infection are present in the region requiring surgery, then cytokines such as tumor necrosis factor and interleukin-6 will accelerate coagulation. 32 33 Antithrombin complexes with the generated thrombin and is rapidly consumed during surgery, contributing to a degree of hypercoagulability proportional to the extent of tissue trauma. 34 Surgery also affects the levels of fibrinolytic factors acutely. Tissue plasminogen activator levels rise in the first few hours but decrease toward normal by 24 hours, and levels of PAI-1 rise fivefold within 2 hours then gradually decrease over 1 to 2 days with a secondary peak at 7 days; the overall effect is a temporary impairment of fibrinolysis. 32 33 35 36 The levels of microparticles also increase in response to surgery and trauma, and these microparticles are recruited into areas of developing clot, contributing to its propagation. 37

Medications administered during surgery, ambient conditions, and surgical techniques also variably affect hemostasis. For example, anesthetic agents may induce stasis and venodilatation, which increase thrombosis risk, 38 39 whereas some agents increase the bleeding risk ( Table 3 ). Prolonged severe hypothermia (<32°C) and acidosis both impair hemostasis and should be optimized in any bleeding patient. 40 These conditions also impact on coagulation testing and may produce abnormal laboratory test results in the absence of any specific factor deficiency. Hetastarch has been associated with increased post–coronary artery bypass grafting hemorrhage, but no differences in hemostasis were seen with the use of normal saline or Ringer's lactate. 41 Use of a tourniquet in total knee replacement increases fibrinolysis and reduces hypercoagulability. 42

**Table 3:** Drugs that affect hemostasis

	Drug	Drug target/receptor(s)
Drugs that affect platelet function		
Intentional	Aspirin	COX-1
	Clopidogrel, ticlopidine, prasugrel, ticagrelor, cangrelor	P2Y12 receptor
	Abciximab, eptifibatide, tirofiban	GP IIb/IIIa (fibrinogen receptor)
	Dipyrdamole	PDE
	Cilostazol	PDE3
	Vorapaxar	PAR1
Incidental	Many drugs and supplements, for example, NSAIDs: meclofenamic acid, mefenamic acid, diclofenac, ibuprofen, indomethacin, naproxen, tolmetin, zomepirac, piroxicam, diflunisal, sulindac	COX-1 (reversible inhibition)
	Antibiotics (penicillins and cephalosporins)	Interaction with platelet receptors, membrane constituents and/or VWF
	Cardiovascular drugs (nitrates, beta-adrenergic receptor blockers, calcium channel blockers, angiotensin-converting enzyme inhibitors, angiotensin receptor blockers, antiarrhythmic drugs)	Various mechanisms
	Lipid-lowering drugs (HMG-CoA inhibitors [statins])	Changes in lipid composition of the platelet plasma membrane; inhibition of GTP-binding proteins?
	Plasma expanders (dextrans, hydroxyethyl starch)	Interaction with platelet membrane constituents
	Antihistamines, radiographic contrast agents, psychotropic drugs (tricyclic antidepressants, phenothiazines, selective serotonin reuptake inhibitors), chemotherapeutic agents, anesthetics and narcotics,	Various mechanisms
	Foods, spices, vitamins and herbal supplements (ginger, onion, vitamin E, cumin, turmeric, cloves, alcohol, omega-3 fatty acids, Chinese black tree fungus, garlic, berries, caffeine, cocoa, dark chocolate, kiwi fruit, purple grape juice, red wine, white wine, tomato, turmeric/curcumin, andrographis, cranberry, danshen, dong quai, feverfew, ginkgo, ginseng, green tea, hawthorn, motherwort, St. John's wort, turmeric, willow bark)	Various mechanisms
Drugs that affect coagulation		
Intentional	UFH, LMWH, lepirudin, argatroban, bivalirudin, dabigatran	Thrombin
	UFH, LMWH, fondaparinux, danaparoid, apixaban, rivaroxaban, edoxaban	Xa
	Vitamin K antagonists (e.g., warfarin)	Factors II, VII, IX, and X
	Thrombolytic agents (streptokinase, urokinase, t-PA)	Fibrin/fibrinogen
	Antifibrinolytic agents (E-aminocaproic acid, tranexamic acid) a	Fibrinolysis
Incidental	Super-warfarins (“rat poison”)	Factors II, VII, IX, and X
	Foods, spices, vitamins and herbal supplements (ginseng, danshen, dong quai, St. John's wort, cranberry)	Various mechanisms
	Plasma expanders (dextrans, hydroxyethyl starch)	

Abbreviations: COX, cyclooxygenase; HMG-CoA, 3-hydroxy-3-methylglutaryl-coenzyme A; GP, glycoprotein; GTP, guanosine triphosphate; LMWH, low-molecular-weight heparin; NSAIDs, non-steroidal anti-inflammatory drugs; PAR, protease-activated receptor; PDE, phosphodiesterase; t-PA, tissue-type plasminogen activator; UFH, unfractionated heparin; VWF, von Willebrand factor.

Note: This list is not meant to be exhaustive, but contains some common as well as less well-recognized agents that affect hemostasis. Summarized from various references, especially references 67 68 69 .

a Unlike all other agents listed in this table, antifibrinolytic agents do not increase bleeding risk.

Some surgical procedures are associated with particular changes in hemostatic function that require specific knowledge for recognition and appropriate preventative action to avoid excessive bleeding. Cardiopulmonary bypass surgery is an example of surgery associated with complex hemostatic changes. Activation of hemostasis occurs while on bypass, and heparin is routinely used to prevent thrombosis; however, its use in combination with inflammation and hemodilution also contributes to blood loss. 43 Other contributors to bleeding may be deficiency of fibrinogen, thrombocytopenia or impaired platelet function, reduced generation of thrombin, hyperfibrinolysis, or surgical bleeding. 43 44 Endothelial disruption and consumption of antithrombin potentially contribute to thrombotic risks. 44 Thromboelastography, a whole blood point of care assay that assesses viscoelastic clot properties, has been used in this setting to distinguish the factors contributing to bleeding and guide replacement of prohemostatic agents or drug therapies including protamine to reverse the effect of heparin, antifibrinolytics, platelet transfusions, administration of coagulation factor concentrates, and cryoprecipitate or fibrinogen. 43 44

## What Causes Bleeding and How Can Different Causes Be Recognized?

Excessive bleeding, greater than expected by the surgeon, is reported in ∼3% of all procedures. 45 In 75 to 90% of cases, intraoperative and early postoperative bleeding result from a technical defect. 46 47 Although arterial and venous bleeding requires surgical intervention with cautery, pressure, ligature, or tamponade for hemostasis, control of bleeding in the microcirculation requires an intact hemostatic system. Thus, distinguishing the different causes for bleeding is crucial, and the clinical features may provide some clues. A structural defect requiring surgical intervention is more likely to be the cause of bleeding that occurs from a single site, is of sudden onset, and/or represents a massive hemorrhage or pulsatile bleeding where a source is evident. A hemostatic defect is the more likely cause when bleeding occurs at multiple sites simultaneously, such as the surgical site in addition to vascular access sites and mucous membranes, skin, or nonoperative hematuria. Hemorrhage that is delayed and occurs after a period of adequate hemostasis or a slow persistent ooze of blood without an obvious source also suggests a defect in hemostasis. 46 47 For example, previously undiagnosed mild hemophilia is a rare cause of surgical bleeding but typically is associated with normal hemostasis intraoperatively and in the initial postoperative period when large amounts of TF are present due to tissue injury. Subsequently, when TF levels fall, a slow but persistent ooze commences between days 1 and 3 postoperatively. 48

Intraoperative hemorrhage is most commonly caused by structural defects, anticoagulant excess, hyperfibrinolysis, or a generalized and severe disorder of hemostasis, such as disseminated intravascular coagulation. Early postoperative hemorrhage (within 2 days of surgery) suggests a defect in primary hemostasis, such as significant thrombocytopenia (platelet count less than 50 × 10 ^9^ /L) or platelet dysfunction, each of which may be inherited or acquired. Mild to moderate inherited disorders of coagulation, such as VWD, may present with early bleeding. Delayed postoperative hemorrhage (onset between days 2 to 7) may also be seen with these causes and in addition with defects in secondary hemostasis, involving coagulation factors, fibrinogen, or connective tissue disorders. Examples of the conditions associated with delayed hemorrhage include vitamin K deficiency, multiorgan failure, recommencement of antiplatelet agents, or anticoagulation, and it has also been reported with the development of antibodies to FV or thrombin associated with bovine thrombin used in fibrin glue. 49 50 Thus, the time frame of bleeding onset gives an indication of likely causes for bleeding. Potential hereditary or acquired causes are listed in Table 2 .

In liver disease, the situation is more complex. Recent theory suggests that hemostasis has been “rebalanced” in patients with liver disease compared with healthy individuals, which has important implications for perioperative management. 51 52 53 54 55 Liver synthesis of coagulation factors is reduced, which results in prolongation of the PT and APTT, which in turn can lead to the misconception that these patients are autoanticoagulated or have a tendency to bleed similar to that seen in patients taking vitamin K antagonists with comparable coagulation assay prolongation. In fact, in liver disease, the normal synthesis of anticoagulant proteins antithrombin, protein C, and protein S is also impaired, providing a counterbalance to the reduced coagulation factors (which is not detected by routine coagulation assays). The liver also synthesizes some components of the fibrinolytic system and is responsible for their clearance, consequently some studies have shown a hyperfibrinolytic state in liver disease. 51 52 53 54 55 Furthermore, the production of thrombopoietin, which stimulates the production of megakaryocytes (the bone marrow precursor to platelets), is impaired and thus contributes to thrombocytopenia, as does platelet sequestration within the enlarged spleen due to portal hypertension. This condition is partially compensated by increased VWF due to reduced liver clearance and possibly increased endothelial production and/or release. Thus, the rebalanced hemostatic state is not reflected by the results of routine APTT and PT assays. Although maintaining a platelet count above 50 × 10 ^9^ /L has been shown to reduce perioperative bleeding, prophylactic preprocedure correction of PT and APTT has not. Similarly, liver disease does not protect patients from thrombotic events, and venous thromboembolism prophylaxis should not be withheld. 51 52 53 54 55

## How Can a Surgeon Predict Perioperative Bleeding?

It is not always possible to predict whether an individual patient will bleed excessively during surgery or postoperatively. However, taking a patient's history and physical examination targeted toward detection of hemostatic defects are by far the best aids in predicting surgical bleeding. 56 57 58 59 Three large studies examined preoperative hemostatic testing and found no correlation between preoperative coagulation screening tests and surgical bleeding due to a preponderance of sample collection or handling errors, laboratory errors, or the presence of “apparent hemostasis abnormalities” such as a lupus anticoagulant or FXII deficiency, which prolong APTT but are not associated with clinical bleeding. 60 61 62 Of 4,499 patients, 85 bled during surgery, 70% of whom had normal coagulation tests. Of the 435 patients with abnormal coagulation tests, 97% did not bleed. The coagulation screening tests showed a sensitivity of 18% for surgical bleeding and specificity of 90% with a positive predictive value of only 3% and negative predictive value of 98%. A history of bleeding was 12.5 times more likely to predict bleeding during subsequent surgery than laboratory screening tests. 63 Furthermore, up to two thirds of patients with abnormal screening tests when investigated further did not have a bleeding diathesis and were not at risk of surgical bleeding. 64 Consequently, testing should be utilized to further evaluate patients whose history indicates a possible bleeding diathesis and for those undergoing major surgery, particularly surgery with high bleeding risk such as procedures involving the central nervous system, cardiopulmonary bypass surgery, or prostatectomy. 47 65

Inappropriate testing or screening all surgical candidates without regard for clinical history increases the risk that statistical, preanalytical, and analytical errors will produce false-positive results. 66 These results will lead to further invasive investigations, increased patient anxiety, and greater utilization of health care resources including laboratory staff time, laboratory resources, and clinical staff time to obtain an appropriate history and try to determine the significance of screening results, as well as potentially delaying surgery unnecessarily. Normal screening tests may also provide false reassurance that a patient is not at risk of bleeding, because many bleeding disorders are not detected by routine screening tests, as discussed above.

When taking a history to screen for abnormal hemostasis, the following questions should be addressed: Has the patient or any family member ever experienced abnormal bleeding or been described as a “bleeder”? Has the patient bled with previous surgery, procedures, dental extractions, or childbirth? Did the bleeding require return to the operating theater for hemostatic control, or did it necessitate blood transfusion or transfusion of other products? Has the female patient had menorrhagia? If so, this finding should be quantified and the requirement for iron supplementation or treatment of anemia questioned. A history of spontaneous bruising at multiple sites, recurrent spontaneous epistaxis, and hemorrhage following trauma or sport should also be sought. If the patient has ever required blood or plasma transfusion, then the reason should be determined. More detailed questioning or hematologist consultation may be advised if these screening questions indicate a possible underlying bleeding disorder.

The preoperative history should also evaluate the patient's medications and recent drug and supplement intake. Many drugs and supplements affect hemostasis, including platelet function and coagulation ( Table 3 ). 67 68 69 Apart from the more obvious (intentionally prescribed) anticoagulant/antithrombotic/antiplatelet agents, the history should also evaluate other recently ingested agents. For example, many herbs and supplements used in traditional Chinese medicine have antihemostatic effects, many drugs affect platelet function, and even some common pain killers (including nonsteroidal anti-inflammatory agents) can potentially increase the risk of bleeding.

## How Can the Cause of Bleeding Be Diagnosed?

**Fig. 4:**
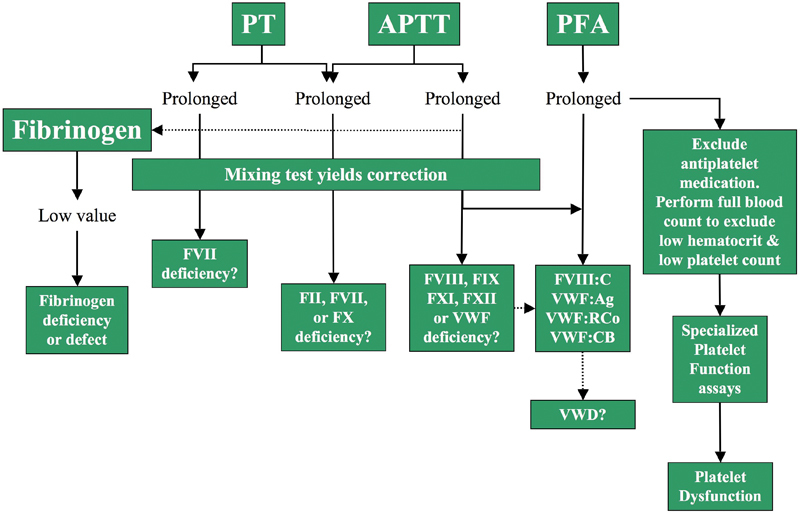
An algorithm that describes a simple approach to the initial investigation of a bleeding patient, or to otherwise screen for hemostasis defects, where simple mixing studies correct and suggest a factor deficiency. Abbreviations: APTT, activated partial thromboplastin time; F, factor; PFA, platelet function analyzer; PT, prothrombin time; VWD, von Willebrand disease; VWF, von Willebrand factor; C, coagulant; Ag, antigen; RCo, ristocetin cofactor; CB, collagen binding.

**Fig. 5:**
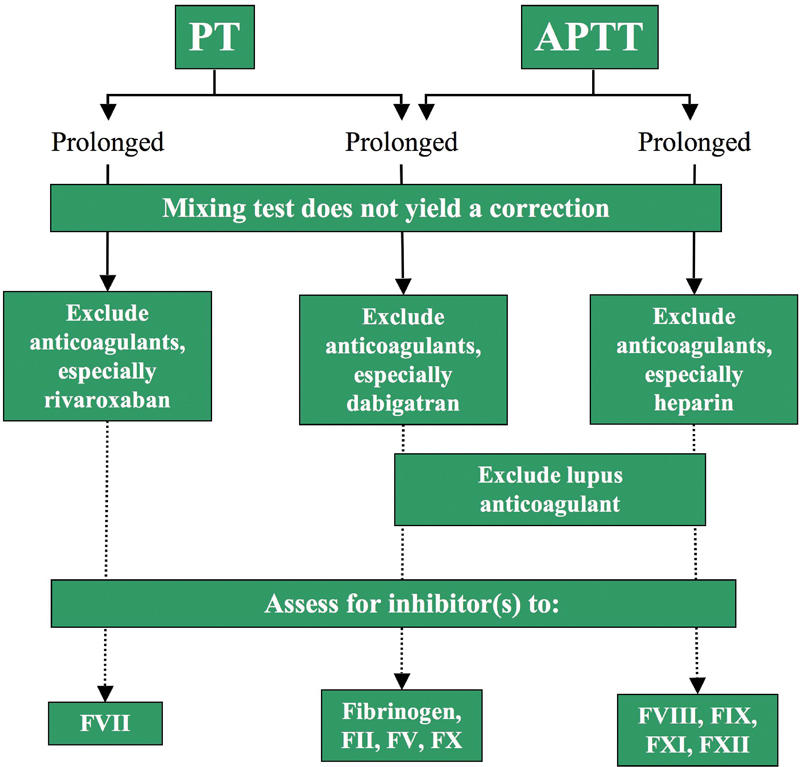
An algorithm that describes a simple approach to the initial investigation of a bleeding patient, or to otherwise screen for hemostasis defects, where simple mixing studies do not correct and suggest the presence of an inhibitor. Abbreviations: APTT, activated partial thromboplastin time; F, factor; PT, prothrombin time.

Although a history of bleeding is invaluable in distinguishing different causes of hemostatic defects, it is not always available in the acutely bleeding surgical patient, who may never have had such a challenge of hemostatic function previously. Urgent investigation and empiric therapy may be required. A fresh peripheral venipuncture should be used for urgent blood sampling to avoid contamination by infused products or anticoagulants. A full blood count, APTT, PT, thrombin time, fibrinogen, and D-dimer should be requested urgently. 70 If available, a platelet function screen using a platelet function analyzer-100/200 may further inform, especially if platelet count and hematocrit are normal. 71 Liaison with the hematologist will enable close collaboration with the coagulation laboratory to ensure appropriate sequential testing. Discussion with the hematologist about the type of bleeding observed and any pre-existing risk factors such as abnormal screening coagulation tests, use of anticoagulants, or antiplatelet therapy will also assist in making a specific diagnosis. Mixing studies and specific coagulation factor testing or VWD screening may be expedited if indicated by the initial tests ( Figs. 4 and 5 ). 70 It may be necessary to exclude an acquired inhibitor. Table 4 indicates likely patterns of abnormal coagulation test results in patients with specific disorders of hemostasis. Attention should also be given to pre-existing abnormal liver or renal function, anesthesia time, patient temperature, ionized calcium concentration, and pH. Abnormalities should be corrected, and urgent empiric therapy may be initiated. Figs. 4 and 5 , respectively, provide further guidance in the situations where mixing studies of initially abnormal PT and/or APTT tests correct (thus suggesting a factor deficiency) or do not fully correct (thus suggesting an inhibitor).

**Table 4:** Laboratory profile for disorders of hemostasis that cause bleeding

Disorder	Platelet count	Platelet morphology	APTT	PT	Mixing studies	Fibrinogen level	TT	D-dimer	Specialized tests required
ITP	⇓	N or Abn	N	N	N	N	N	N	
Hereditary platelet disorders	⇓ or N	N or Abn	N	N	N	N	N	N	Platelet aggregation studies
VWD	N (or ⇓; 2B)	N	N (or ⇑)	N	N	N	N	N	von Willebrand studies
Hypofibrinogenemia	N	N	N (or ⇑)	N (or ⇑)	N	⇓	⇑	N	Special fibrinogen assays
Dysfibrinogenemia	N	N	N (or ⇑)	N (or ⇑)	N	⇓	⇑	N	Special fibrinogen assays
DIC	N	N	N (or ⇑)	N (or ⇑)	N	⇓ (or N)	⇑	⇑	
Factor VIII, IX, or XI deficiency	N	N	⇑	N	N	N	N	N	Specific factor assays
Factor VII deficiency	N	N	N	⇑	N	N	N	N	Specific factor assays
Factor II, V, or X deficiency	N	N	⇑	⇑	N	N	N	N	Specific factor assays
Factor XIII deficiency	N	N	N	N	N	N	N	N	Specific factor assays
Liver disease	N	N	⇑ (or N)	⇑	N	⇓ (or N)	N	N	Specific factor assays
Vitamin K deficiency/warfarin therapy	N	N	⇑ (or N)	⇑	N	N	N	N	Specific factor assays
Dabigatran anticoagulation	N	N	⇑ (or N)	N (or ⇑)	Abn	N	⇑	N	Specific drug assay
Oral direct Xa inhibitor anticoagulants	N	N	N (or ⇑)	⇑ (or N)	Abn	N	N	N	Specific drug assay
Connective tissue disorders	N	N	N	N	N	N	N	N	Specific assays

Abbreviations: ⇓, decreased compared to normal; ⇑, increased compared to normal; Abn, abnormal; APTT, activated partial thromboplastin time; DIC, disseminated intravascular coagulation; ITP, immune thrombocytopenia; N, normal; PT, prothrombin time; TT, thrombin time; VWD, von Willebrand disease.

For example, VWD is considered the most common inherited bleeding disorder with an estimated prevalence up to 1%; it is due to deficiency and/or defects in the plasma protein VWF. 29 72 73 VWD is classified on the basis of quantitative deficiencies of VWF (VWD types 1 and 3) or qualitative defects in VWF (type 2 VWD), which may or may not be also associated with a quantitative deficiency of VWF. Bleeding in VWD is typically of the mucocutaneous type with menorrhagia, epistaxis, gum bleeds, or gastrointestinal bleeds reflecting the function of VWF in adhesion of platelets and subendothelial matrix. A further consequence of the reduced, absent, or dysfunctional VWF is loss of the protective function of plasma FVIII against proteolysis. Thus, the coassociated deficiency of FVIII will also contribute to bleeding events in a secondary picture that is more typical of hemophilia A with muscular hematomas, joint bleeds, or postsurgical bleeding from large wounds. Treatment replaces both missing components, VWF and FVIII.

Disorders of platelet function should be investigated if the bleeding looks mucosal in origin but VWD testing is normal. 74

For patients already known to have a bleeding disorder, preoperative assessment by a hematologist should be undertaken to optimize the perioperative management of the disorder. In more severe bleeding disorders, surgery should be performed in a setting with appropriate medical, nursing, and laboratory supports, such as a hemophilia treatment center.

Deficiency of FXII is a more common cause of APTT prolongation than mild hemophilia. Deficiencies of contact factors (FXII, high-molecular-weight kininogen, and prekallikrein) do not require replacement for surgery but will cause prolongation of the baseline APTT and activated clotting time; consequently, these assays are unsuitable for monitoring anticoagulant therapy in some patients, for example, those having cardiac surgery. Such cases should be discussed with the hematologist and alternative assays such as anti-Xa activity or thrombin generation may be available.

## How Should Unexpected Bleeding due to Hemostatic Defects Be Treated?

Urgent resuscitation should be instituted without delay, including acute volume replacement and red cell transfusion if indicated. Massive transfusion protocols for major bleeding usually recommend proportional replacement of red cells, platelets, fresh frozen plasma, and cryoprecipitate to prevent coagulopathy due to dilutional losses of platelets and coagulation factors. If bleeding decreases following these measures, then it may be appropriate to watch and wait while additional coagulation tests are conducted. If bleeding continues, then re-exploration of the surgical bed or bleeding site may be indicated. In some circumstances, specific procedures such as angiography with or without arterial embolization may be indicated. Infusion of desmopressin (DDAVP ^®^ , Ferring Pharmaceuticals Ltd, West Drayton, United Kingdom; 0.3 μg/kg in 50 mL normal saline over 30 minutes) produces a two- to fivefold increase from baseline in VWF levels in responders and may also improve platelet function in uremic patients. 75 76 By 24 hours, levels generally return to baseline. 72 77 Doses may be repeated up to 72 hours but responses diminish due to depletion of VWF stores, described as tachyphylaxis. Adverse effects include facial flushing, hypertension or hypotension, tachycardia, headache, gastrointestinal upset, and hyponatremia, rarely complicated by seizures. Myocardial infarction has rarely been reported, and thus desmopressin should be avoided in patients with increased risk for cardiovascular and cerebrovascular disease. 72 Fluid restriction and monitoring of electrolytes is recommended in all cases, particularly when repeat doses are used. In cases of suspected VWD and where DDAVP response is inadequate or contraindicated, treatment entails replacing the missing VWF and FVIII, aiming to increase levels to normal ( Table 5 ).

**Table 5:** A summary of current therapies for major bleeding or surgery in patients with hereditary disorders of hemostasis

Disorder	Main therapies	Initial dose in major surgery/bleeding	Initial therapeutic target for treatment of major bleeding
VWD type 1	DDAVP VWF(/FVIII) concentrate	DDAVP 0.3 μg/kg 40–60 U/kg in VWF:RCo IU/dL	Trough VWF:RCo and FVIII > 50 IU/dL
VWD type 2A, 2M, 2N	VWF(/FVIII) concentrate DDAVP	40–60 U/kg in VWF:RCo IU/dL 0.3 μg/kg	Trough VWF:RCo and FVIII > 50 IU/dL
VWD type 2B, type 3	VWF(/FVIII) concentrate	40–60 U/kg in VWF:RCo IU/dL	Trough VWF:RCo and FVIII > 50 IU/dL
Hemophilia A	FVIII concentrates	Dose varies with severity (1 U/kg raises plasma level by 2 U/dL)	FVIII 80–100 IU/dL
Hemophilia B	FIX concentrates	Dose varies with severity (1 U/kg raises plasma level by 1 U/dL)	FIX 80–100 IU/dL
Hypofibrinogenemia, dysfibrinogenemia	Cryoprecipitate Fibrinogen concentrate	15–20 mL/kg 50–100 mg/kg	Fibrinogen 1 g/L or higher
Prothrombin deficiency	Fresh frozen plasma PCCs	15–25 mL/kg 20–40 U/kg	>20 IU/dL
Factor V deficiency	Fresh frozen plasma	15–25 mL/kg	>15–20 IU/dL
Factor VII deficiency	FVII concentrate if available PCC rFVIIa	30–40 mL/kg 20–30 U/kg 15–30 μg/kg every 4–6 h	>20 IU/dL
Factor X deficiency	Fresh frozen plasma PCC	10–20 mL/kg 20–30 U/kg	>20 IU/dL
Factor XI deficiency	Fresh frozen plasma FXI concentrate	15–20 mL/kg 15–20 U/kg	15–20 IU/dL
Factor XIII deficiency	Cryoprecipitate rFXIII if available	2–3 units 35 U/kg	30%
Ehlers-Danlos syndrome	No specific therapy; ascorbic acid supplementation or DDAVP perioperatively		

Abbreviations: F, factor; PCC, prothrombin complex concentrate; rF, recombinant factor; VWD, von Willebrand disease; RCo, ristocetin cofactor.

Note: Summarized from several references, especially references 91 92 93 94 95 .

Adjunctive therapies such as antifibrinolytics should also be considered. Bleeding is more likely to occur with oral procedures and prostate or urologic surgery due to the presence of the natural profibrinolytic urokinase in saliva and urine, which will dissolve hemostatic clots in wounds. Tranexamic acid (20 mg/kg orally or intravenously every 8 hours) or ε-aminocaproic acid (2 g orally every 8 hours) will inhibit plasmin generation and prevent excessive fibrinolysis.

Patients with specific inhibitors of coagulation factors, especially FVIII, whether known or unexpected, are at particularly high risk of bleeding. Activated prothrombin complex concentrates such as FVIII inhibitor bypassing agent may be effective and is typically given as an initial bolus of 100 U/kg followed by 50 U/kg every 8 to 12 hours. 78 79 Recombinant FVIIa is useful in patients with inhibitors to FVIII or FIX and has been used in FXI deficiency but has been associated with increased risk of thrombosis when used in the setting of trauma or unexplained surgical hemorrhage, without evidence of mortality benefit. 80

Inherited deficiencies of fibrinogen, factor II, FV, combined factor V and VIII, FVII, FX, FXI, and FXIII are collectively referred to as rare bleeding disorders, with predominantly autosomal-recessive inheritance. 81 82 Symptoms vary from mild to severe and may not be evident until a surgical challenge occurs. Correction of deficiency is achieved with fresh frozen plasma, cryoprecipitate, or specific factor concentrates where available. Table 5 summaries specific therapies that are available for the treatment of surgical bleeding once a specific diagnosis has been made or is suspected.

Allogeneic blood components are widely used for surgical bleeding; however, for some patients transfusions are not acceptable on religious grounds. 83 It should be determined preoperatively which blood components and procedures are acceptable to the individual patient. Hematinic deficiencies should be corrected preoperatively to optimize baseline hemoglobin. Blood salvage techniques and local hemostatic agents should be implemented to minimize blood loss. These principles of patient blood management are applicable to all patients and have shown that outcomes can be achieved that are similar to those for patients who do accept transfusions. 83

Additional and alternate strategies may need to be employed in the case where unexpected bleeding is associated with the use of unrecognized or even recognized anticoagulation/antiplatelet therapy. 84 85 86 Principles of managing bleeding include withdrawal of the anticoagulant or antiplatelet agent, supportive care, and administration of a specific antidote if it is available. Vitamin K antagonists, such as warfarin, may be acutely reversed with prothrombin complex concentrate (20 to 50 U/kg) or if contraindicated in the specific patient, by using fresh frozen plasma. Vitamin K is a specific antidote but it takes at least 12 hours to work. 87 Protamine sulfate binds to and inactivates unfractionated heparin and is dosed at 1 mg per 100 U of heparin given in the preceding 2 to 3 hours. It may also be used for low-molecular-weight heparin (1 mg per 100 Xa units given in last 8 hours) but is only partially effective because it binds to the longer chain molecules only. It is ineffective for reversal of the pentasaccharide fondaparinux. 87 The oral direct thrombin inhibitor dabigatran now has a specific antidote, idarucizumab (Praxbind ^®^ , Boehringer Ingelheim, Ingelheim, Germany ), an antibody fragment with a very high affinity for dabigatran that recently received U.S. Food and Drug Administration approval. Interim analysis of the phase III REVERSE-AD trial showed immediate and sustained reversal of the anticoagulant effect of dabigatran as measured by coagulation assays with a 5 g intravenous dose. 88 Patients who received the agent preoperatively for urgent surgery were thought by the operating surgeon to subsequently have normal hemostasis during the procedure in 92% of cases. Andexanet is a specific reversal agent for Xa inhibitors, both oral and parenteral, which is administered as an intravenous infusion, but it is still undergoing phase III trials. Limited data supports the use of prothrombin complex concentrate for major bleeding. 87 Although specific assays are now available to measure plasma concentrations of the direct oral anticoagulants, 89 the drug levels at which surgery may be undertaken safely have not been established. Local hemostatic measures are often sufficient to control bleeding associated with antiplatelet therapies. However, for surgery at critical sites such as intracranial, spinal, or ophthalmologic procedures, platelet transfusion and/or DDAVP (0.3 μg/kg) is often utilized, despite a lack of evidence for their effectiveness for clinical bleeding in this setting. 90

## Conclusions

As highlighted by this narrative review, patients can bleed for a large variety of reasons in the perioperative setting, including surgical and anatomical anomalies/disorders, recent drug intake, or disturbances of hemostasis. We provide guidance on how to screen for these conditions, as well as for investigation of the cause of bleeding and interpreting the relevant laboratory investigations. We also outline several management strategies. The identification and management of any specific bleeding lesion remains crucial. Where local hemostatic control is not possible, then supportive measures as well as specific therapies may be necessary. Although some hemostatic agents may be given empirically, selected urgently performed laboratory tests and consultation with the hematologist may establish a specific diagnosis and enable refinement of the management of bleeding in the perioperative setting. Careful preoperative assessment, to identify patients with a history of bleeding or those having taken any of the large number of antiplatelet and anticoagulant agents now available, will also enable appropriate preoperative drug cessation or hemostatic agent administration to prevent major bleeding. The future may provide further refinement in these strategies, as the newer hemostatic agents become more widely available. 90
